# High-Resolution Geospatial Mapping of Zero-Dose and Underimmunized Children Following Nigeria's 2021 Multiple Indicator Cluster Survey/National Immunization Coverage Survey

**DOI:** 10.1093/infdis/jiad476

**Published:** 2023-10-31

**Authors:** Anne Eudes Jean Baptiste, John Wagai, Susan Hahné, Adeyemi Adeniran, Richard Ipuragboma Koko, Stijn de Vos, Messeret Shibeshi, E A M Sanders, Balcha Masresha, Eelko Hak

**Affiliations:** Country Office, World Health Organization, Abuja, Nigeria; Country Office, World Health Organization, Abuja, Nigeria; National Institute for Public Health and the Environment, Bilthoven, the Netherlands; National Bureau of Statistics, Abuja, Nigeria; Country Office, World Health Organization, Abuja, Nigeria; Groningen Research Institute of Pharmacy, University of Groningen, the Netherlands; African Regional Office, World Health Organization, Brazzaville, Congo; Department of Paediatric Immunology and Infectious Diseases, University Medical Centre Utrecht, the Netherlands; African Regional Office, World Health Organization, Brazzaville, Congo; Groningen Research Institute of Pharmacy, University of Groningen, the Netherlands

**Keywords:** geospatial mapping, Nigeria, underimmunized, zero dose

## Abstract

**Background:**

“Zero-dose” children are those who are without any routine vaccination or are lacking the first dose of the diphtheria, tetanus, and pertussis–containing vaccine. Based on global estimates from the World Health Organization/United Nations Children’s Fund in 2022, Nigeria has the highest number of zero-dose children, with >2.3 million unvaccinated.

**Methods:**

We used data from the 2021 Nigeria Multiple Indicator Cluster Survey/National Immunization Coverage Survey to identify zero-dose and underimmunized children. Geospatial modeling techniques were employed to determine the prevalence of zero-dose children and predict risk areas with underimmunized children at a high resolution (1 × 1 km).

**Results:**

Zero-dose and underimmunized children are more prevalent in socially deprived groups. Univariate and multivariate bayesian analyses showed positive correlations between the prevalence of zero-dose and underimmunized children and factors such as stunting, contraceptive prevalence, and literacy. The prevalence of zero-dose and underimmunized children varies significantly by region and ethnicity, with higher rates observed in the country's northern parts. Significant heterogeneity in the distribution of undervaccinated children was observed.

**Conclusions:**

Nigeria needs to enhance its immunization system and coverage. Geospatial modeling can help deliver vaccines effectively to underserved communities. By adopting this approach, countries can ensure equitable vaccine access and contribute to global vaccination objectives.

## Teaser

Nigeria has the highest number of children missing out on all routine immunization vaccinations—“zero-dose children.” Geospatial models can predict areas with zero-dose and underimmunized children for targeted interventions to achieve the Immunization Agenda 2030 goals.

Over the last 2 decades, mortality and morbidity from vaccine-preventable diseases have been on a decline globally because of concerted efforts in vaccinating children, tracking and controlling outbreaks with laboratory-backed surveillance, strengthening immunization services, and aggressive case management [1]. Additionally, global health partnerships focusing on increasing access to vaccines in low-income countries provided support to countries in vaccine financing, health system strengthening, market shaping, as well as technical and program support [2]. However, systemic weaknesses in routine immunization (RI) programs, suboptimal mass supplemental vaccination campaigns, and the presence of communities that cannot access immunization services due to their remote locations, insecurity, or vaccine hesitancy threaten to derail progress toward the vaccination coverage objectives. Furthermore, the COVID-19 pandemic put a strain on the global health system, limiting the ability to distribute vaccines and vaccinate children.

For example, between 2000 and 2018, the increase in measles vaccination coverage resulted in a 73% global decrease in measles mortality. However, in recent years, pockets of immunity gaps have led to a resurgence in measles cases and deaths and to large outbreaks, as measles is very contagious with a reproduction rate of approximately 12% to 18% [1–3]. In 2021, there were an estimated 128 000 measles deaths globally, mostly among unvaccinated or undervaccinated children aged <5 years [4]. Two-dose measles vaccine coverage ≥95% is required to achieve protection for all and to prevent outbreaks [5].

The World Health Organization defines “zero-dose” children as those not vaccinated with at least 1 routine vaccine. For operational purposes, Gavi defines zero-dose children as those who lack the first dose of the diphtheria, tetanus, and pertussis–containing vaccine (DTP1) [4]. An estimated 58% of zero-dose children live in 10 countries. Among the leading countries for DTP1 zero-dose children in 2022, Nigeria has replaced India, which was leading this group in 2021. The country, affected by conflict, has >2.3 million zero-dose children [6, 7]. Nigeria has been facing significant challenges in reducing the number of zero-dose children. The 2018–2019 National Nutrition and Health/SMART Survey reported that 69.9% and 64.7% of children aged 12 to 23 months were vaccinated with Penta 1 (DTP1, hepatitis B, and *Haemophilus influenzae* type b) and first-dose measles-containing vaccine, respectively [8].

Because measles is highly infectious, its presence in the community serves as an early indicator (the “canary in the coal mine”) of inadequate coverage and gaps in the health system [1, 9, 10]. According to the 2022 estimates of national immunization coverages per the World Health Organization/United Nations Children’s Fund, the number children missing out on a measles vaccine had reached 3 million in Nigeria.

In 2021, Nigeria conducted the Multiple Indicator Cluster Survey/National Immunization Coverage Survey (MICS/NICS) to provide reliable estimates at the state and national levels. Following the 2021 MICS/NICS and by using modeling techniques verified in multiple settings, geomapping of zero-dose and undervaccinated vaccination coverage estimates for Nigeria was produced at a granularity of 1 × 1 km. These very high-definition geocoded estimates were aggregated to map those children at the local government area (LGA)/district level and ward/community level, which are the third and fourth administrative levels in Nigeria, respectively. Mapping zero-dose and underimmunized children is essential for identifying vulnerable populations, tailoring immunization strategies, monitoring progress, and strengthening health systems. This study aims to determine the prevalence of zero-dose children at the LGA level by using the 2 operational definitions of zero-dose vaccinations and to predict risk areas with underimmunized children.

## METHODS

We used the 2021 Nigeria MICS/NICS to identify the number of zero-dose children as defined by Johri et al, as all surviving children aged 12 to 23 months who did not receive DTP1 (ie, did not receive any DPT doses) [10]. For underimmunized children, we used the operational definition by Gavi, where an underimmunized child is classified as one missing the third dose of the DTP vaccine [7].

The 2021 MICS/NICS is a household-based coverage survey that assesses vaccination coverage for vaccine antigens given to children aged 12 to 23 months among other indicators. The 2021 MICS/NICS included a supplemental sample (additional enumeration areas/clusters to the original MICS sample) to allow for reporting of immunization-related indicators at the state level for the 36 states and the Federal Capital Territory, Abuja in Nigeria. Data were collected on electronic tablets with CSPro software between August and December 2021. The sample size was 37 000 households in 1850 clusters. Geospatial covariates were collected at each cluster and were available for all the clusters covered. The vaccination status of children was derived by administering a questionnaire to the mothers of primary caregivers of children aged <5 years to determine whether the children had received the vaccine antigens recommended by the Nigeria Expanded Immunisation Program. Evidence for vaccination was elicited through card evidence or from maternal recall. Studies have demonstrated the reliability of maternal recall in the absence of a vaccination card [11, 12].

### Geospatial Model Fitting, Validation, and Prediction

We fitted a geostatistical model to predict the prevalence of zero-dose children in Nigeria using the stochastic partial differentiated equation approach. We used data from the 2021 Nigeria MICS/NICS to identify the children who were zero dose and generated a binary variable for each surviving child aged 12 to 23 months, which was 1 when the child had not received any DPT doses and 0 when the child had received at least 1 dose. We also used publicly available, high-resolution covariates on contraceptive prevalence, stunting in children aged <5 years, literacy rates, night-time lights, and distance from health facility. The choice of covariates was informed by previous work on the use of geostatistical covariates to estimate vaccination coverage [13–17]. We created a triangulated mesh for Nigeria, a projection matrix, and data stacks to fit the model. We then projected the prevalence of zero dose and the 95% CIs. Finally, the prevalence of zero dose was reported at the LGA level by aggregating the means of predicted zero doses in each square kilometer in all LGAs.

To model and predict the prevalence of zero dose at a resolution of 1 × 1 km, we fitted geostatistical models with binomial likelihoods. For i=1,…,n and a given indicator, where *n* is the number of survey locations, let $Y(s_{i})$ denote the number of zero-dose children at survey cluster $\left(s_{i}\right)$ and $m(s_{i})$ the number of children sampled at the location. The first level of the model assumes that


$$Y(s_{i}|\;m(s_{i}),p(s_{i})∼Binomial(m(s_{i}),p(s_{i})),$$

where $p(s_{i})(0\leq p(s_{i})\leq 1)$ is the true vaccination coverage at location $s_{i}$. We model $p(s_{i})$ using the logistic regression model as


$$\begin{array}{r} \mathrm{logit}(p({\text{s}}_{\text{i}}))=\beta_{0}+\beta_{1}\mathrm{contr}a_{\mathrm{previ}}+\beta_{2}{\mathrm{literacy}}_{\mathrm{yi}}\\ +\beta_{3}stunting_{i}+\omega (s_{i})+\varepsilon (s_{i}), \end{array}$$

where $\beta_{0}$ denotes the intercept and $\beta_{1},\beta_{2}and$$\beta_{3}$ the coefficient for contraceptive prevalence, literacy, and stunting and where $\left(s_{i}\right)$ is a spatial random effects variable. With the fitted models, we obtained predictions at a resolution of 1 × 1 km. Furthermore, using the posterior samples of the 1 × 1–km predictions, we obtained the LGA- and state-level predictions as population-weighted averages taken over the 1 × 1–km grid cells falling within each LGA or state. All analyses were carried out in R software and through the R-INLA statistical package.

### Model Validation

The geostatistical model was validated via *k*-fold cross-validation by splitting observations between the training and validation sets and determining how well the model could predict the outcome of validation (unseen) data. Observed and predicted prevalence at the cluster level was compared. The cross-validation was based on a 10% subset of a randomly selected cluster location (*m*). For all the excluded points, we compared the predicted and measured values and computed the percentage bias as $100\times \sum_{i}\left({\hat{p}}_{i}-p_{i}\right)/\sum_{i}p_{i}$ and the validation mean square error as $\sum_{i}{\left({\hat{p}}_{i}-p_{i}\right)}^{2}/m$. We also computed the Pearson correlation between observed coverage (from survey data) and predicted coverage (from the model prediction).

## RESULTS

Table 1 shows the distribution of the prevalence of zero-dose and underimmunized children aged 12 to 23 months by state, geopolitical zone, mothers’ education, and wealth index. Zero-dose and underimmunized children are prevalent in socially deprived groups, such as mothers with little or no education and households in the lowest socioeconomic strata, as compared with children from higher socioeconomic strata. Univariate relationships between zero-dose prevalence and different factors with 95% CIs revealed that the prevalence of zero dose was positively correlated with the prevalence of stunting, contraceptive prevalence, and literacy. Similar risk factors were seen for the prevalence of underimmunized children. Multivariate bayesian analysis also demonstrated a positive correlation between these parameters and the prevalence of zero-dose and underimmunized children.

**Table 1.** Prevalence of Zero-Dose Children (Aged 12–23 Months) by Selected Sociodemographic and Characteristics, Nigeria, 2021

	Zero Dose		Underimmunized	
	%	95% CI	%	95% CI
National	30.1	27.9–32.4	43.9	41.5–46.3
State				
Abia	11.8	5.7–22.8	25	16.4–36.3
Adamawa	25.7	18.2–35.1	46.1	36.7–55.7
Akwa Ibom	13.9	7.4–24.4	26.9	17.3–39.2
Anambra	18.5	3.9–55.9	28.6	10.3–58.1
Bauchi	57.7	44.1–70.2	69.7	58.7–78.9
Bayelsa	23.5	13.8–37.1	31.7	21.1–44.7
Benue	20.8	11.3–35.2	37.4	28.2–47.6
Borno	42.7	28.8–57.9	69.9	58.6–79.2
Cross River	7.5	1.4–31.0	28.8	18.6–41.7
Delta	19.3	9.4–35.5	31	19.5–45.5
Ebonyi	1	.2–3.8	1.3	.3–4.9
Edo	9.4	3.5–22.9	16.9	8.8–30.1
Ekiti	4.5	.9–19.7	17	9.3–29.0
Enugu	1.5	.5–4.4	9.2	4.6–17.5
Gombe	44.9	34.5–55.7	61.7	49.1–73.0
Imo	5.9	1.9–17.0	11.2	4.3–25.8
Jigawa	38.6	30.8–46.9	51.2	41.6–60.7
Kaduna	29.3	21.0–39.2	40.1	29.7–51.6
Kano	45	35.5–54.9	58.4	49.2–67.0
Katsina	47	36.5–57.8	58.8	48.6–68.3
Kebbi	41.1	32.1–50.8	46.5	37.4–55.8
Kogi	13.8	7.2–25.0	38	28.4–48.6
Kwara	33.7	18.1–54.0	44.9	28.9–62.0
Lagos	7.6	4.1–13.9	15	9.7–22.3
Nasarawa	27.3	18.5–38.3	45.8	35.9–56.1
Niger	38.4	28.5–49.4	61.2	50.3–71.0
Ogun	29.8	17.6–45.9	56.6	45.0–67.6
Ondo	29.6	17.0–46.2	40	25.7–56.1
Osun	8.1	4.5–14.3	18.8	11.5–29.2
Oyo	23.9	14.5–36.9	41	30.4–52.5
Plateau	21.3	13.5–32.0	35.7	25.0–48.1
Rivers	10.4	5.5–18.7	21.9	13.8–33.0
Sokoto	72.4	59.5–82.4	88.5	79.9–93.7
Taraba	29.9	20.5–41.2	50.5	41.2–59.7
Yobe	26.7	17.2–39.0	35.8	24.1–49.6
Zamfara	55.7	45.9–65.0	75.2	67.1–81.9
Federal Capital Territory Abuja	9.5	4.0–20.8	20.4	11.3–34.0
Geopolitical zone				
North Central	25.4	20.9–30.6	43.3	38.6–48.1
North East	41.6	36.1–47.3	58.8	53.7–63.8
North West	45	40.9–49.1	57.5	53.5–61.4
South East	7.1	3.2–14.7	13.9	8.4–22.2
South-South	13.3	9.8–17.8	25.5	20.9–30.7
South West	16.6	12.6–21.5	30.1	25.4–35.3
Ethnicity of household head				
Hausa	46.6	42.7–50.6	58.8	55.0–62.5
Igbo	6.1	3.2–11.2	12.6	8.5–18.3
Yoruba	17.3	13.2–22.4	31.4	26.5–36.9
Fulani	53.5	46.3–60.6	69	61.9–75.2
Kanuri	34.2	24.0–46.1	57.3	43.9–69.8
Ijaw	22.9	12.4–38.5	32.7	20.5–47.7
Tiv	22	11.6–37.7	39.6	29.4–50.7
Ibibio	12.2	5.4–25.2	21.9	12.7–35.0
Edo	8.9	3.8–19.5	16.5	8.3–30.1
Other ethnicities	21	18.0–24.4	39.2	35.4–43.1
Mother's education				
None	50.9	47.6–54.2	64	60.9–66.9
Primary	30.3	26.1–34.9	47.8	42.8–52.9
Junior secondary	25	19.8–31.1	39.2	33.3–45.4
Senior secondary	12.8	10.5–15.7	27.9	24.6–31.5
Higher/tertiary	3.4	2.1–5.4	11	7.9–15.0
Missing/don't know	24.1	1.9–83.6	24.1	1.9–83.6
Wealth index quintile				
Poorest	48.6	44.5–52.8	61.6	57.5–65.5
Second	40	36.0–44.1	56	52.2–59.7
Middle	26.2	22.7–29.9	42.9	39.0–46.9
Fourth	17.2	13.7–21.4	30.4	25.9–35.4
Richest	7	4.8–9.9	15.6	12.1–19.9

Source: Nigeria 2021 Multiple Indicator Cluster Survey/National Immunization Coverage Survey.

**Figure 1.**
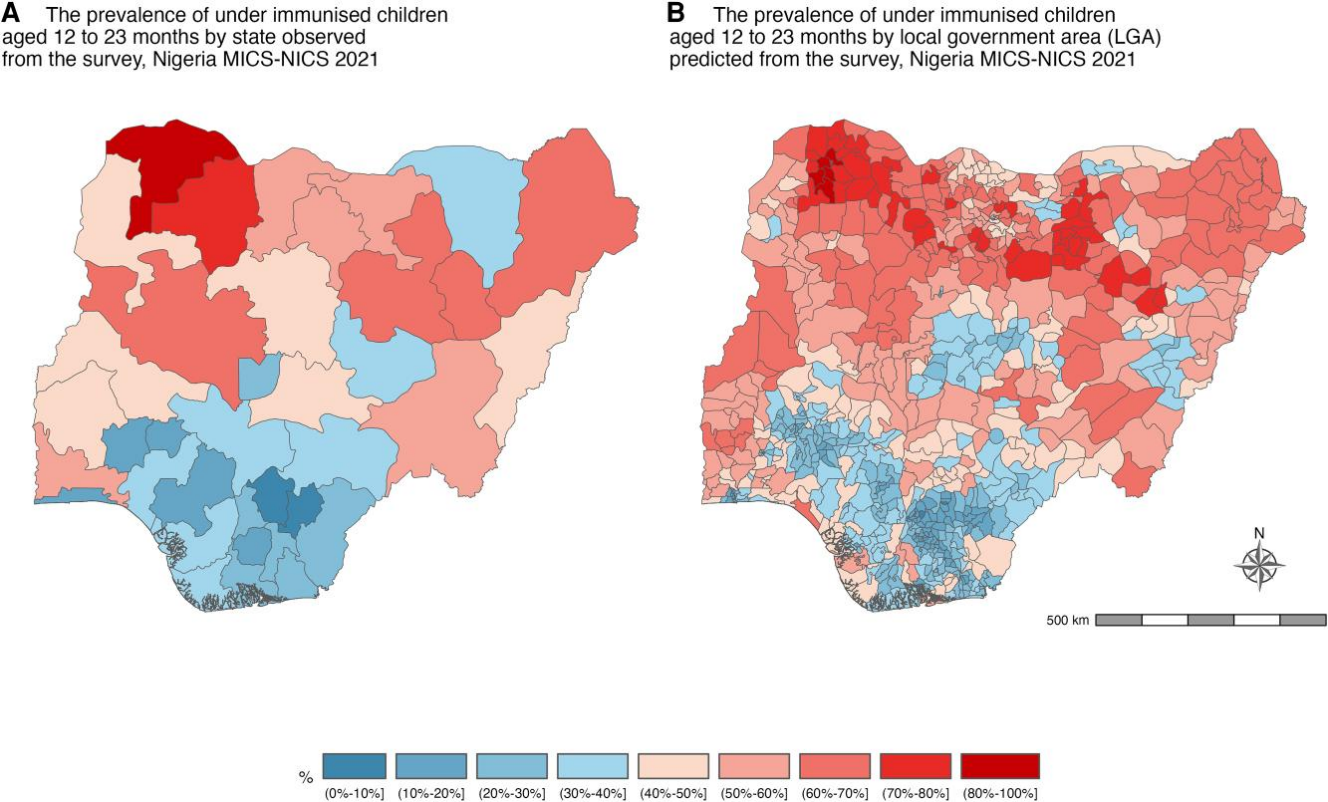
Maps of Nigeria display data from the 2021 Multiple Indicator Cluster Survey/National Immunization Coverage Survey (MICS/NICS). *A* and *B*, Maps illustrate the observed and predicted prevalence of underimmunized children aged 12 to 23 months within Nigeria's states and local government areas. Underimmunized children are those who missed the third dose of the diphtheria, tetanus, and pertussis–containing vaccine.

**Figure 2.**
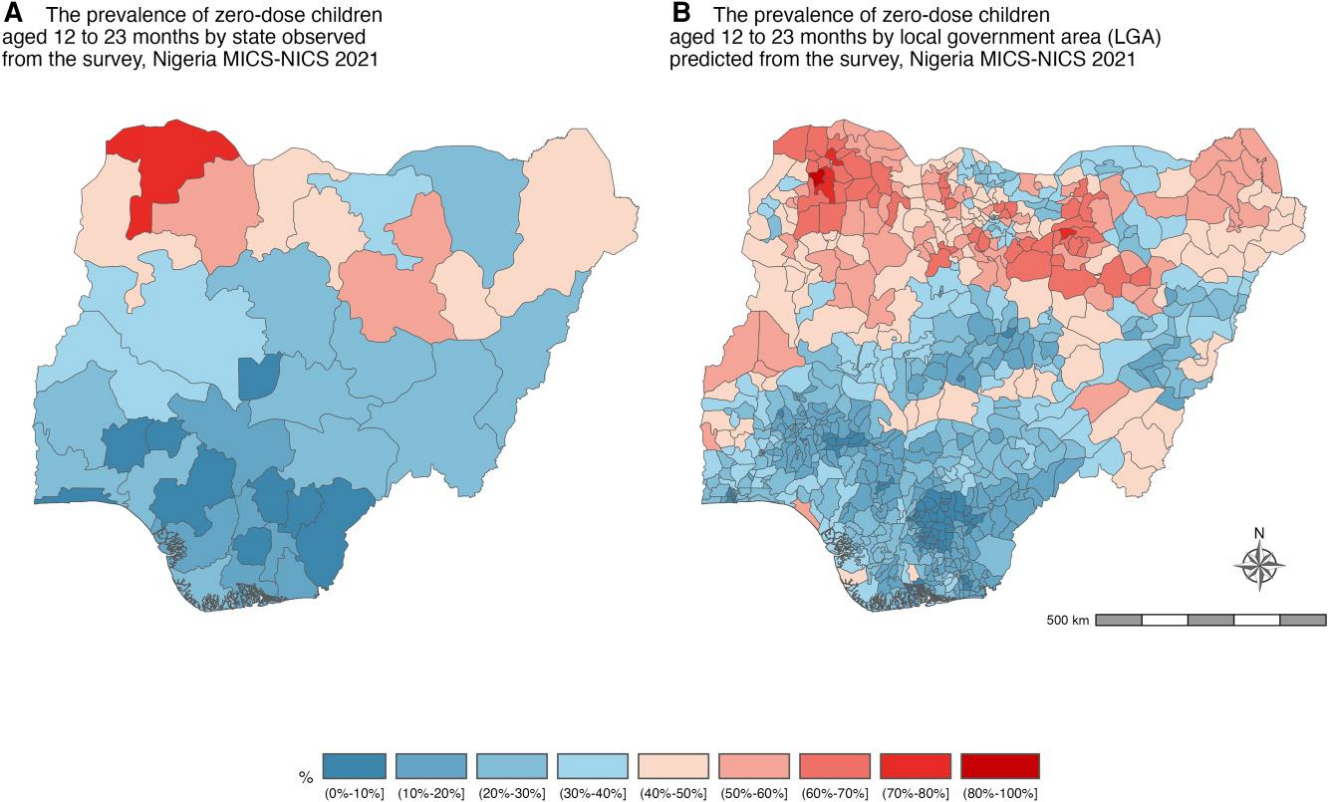
Maps depict the observed and predicted prevalence of zero-dose children: surviving children aged 12 to 23 months who did not receive the initial diphtheria, tetanus, and pertussis–containing vaccine or any dose. *A* and *B*, Maps of Nigeria are based on data from the 2021 Multiple Indicator Cluster Survey/National Immunization Coverage Survey (MICS/NICS), covering all states and local government areas.

The observed and predicted prevalence of underimmunized children aged 12 to 23 months is illustrated in Figure 1, which shows that a significant proportion of children were estimated to be undervaccinated, with large heterogeneity in the distributions. The prevalence of observed and predicted zero-dose children are illustrated in Figure 2. Zero dose children were markedly higher in states in the northern parts of the country. The percentage of underimmunized children was also higher in the northern parts.

**Figure 3.**
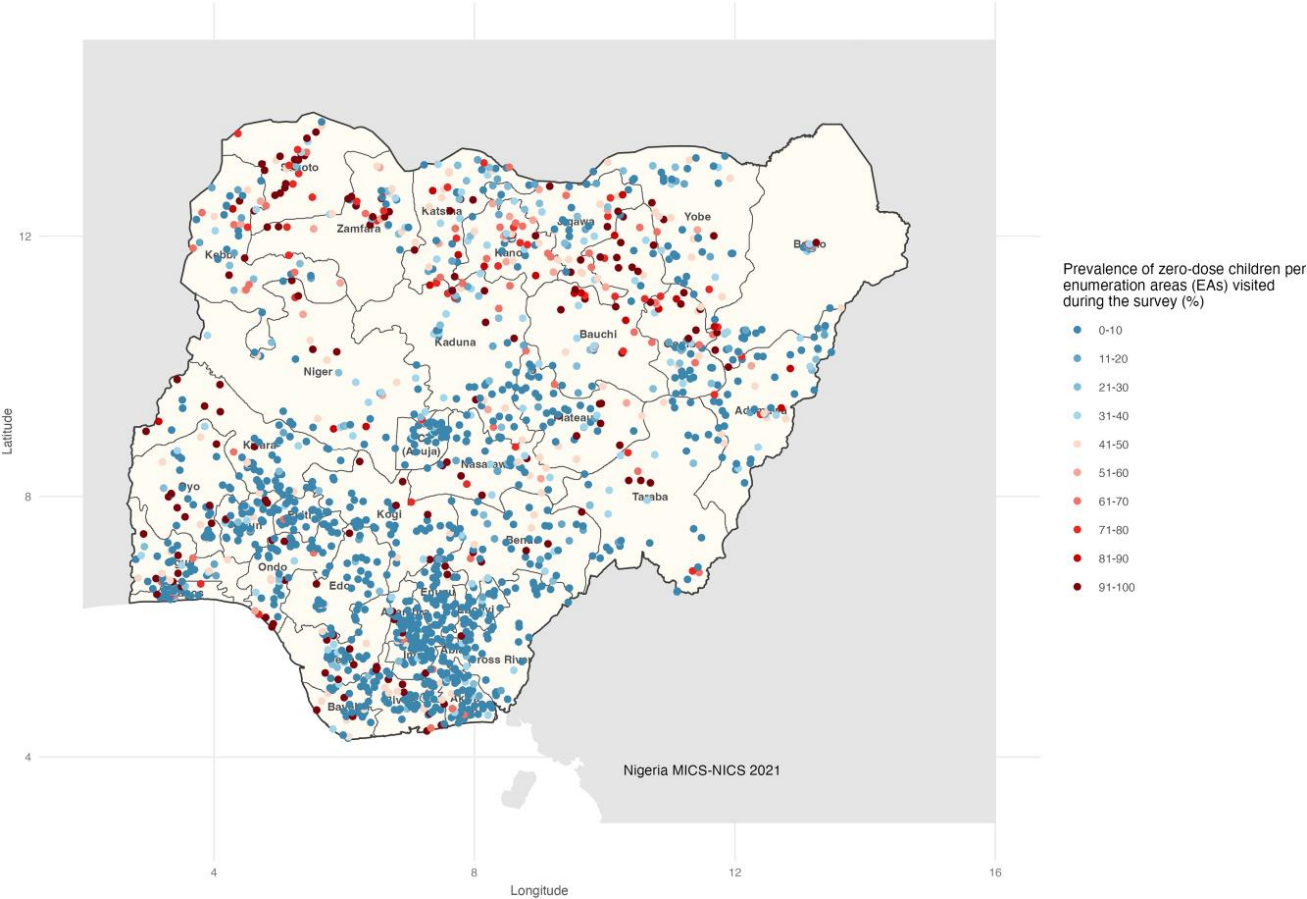
Prevalence of zero-dose children per enumeration area visited during the Multiple Indicator Cluster Survey/National Immunization Coverage Survey (MICS/NICS). For 36 states and the Federal Capital Territory Abuja, 50 enumeration areas (EAs) per state and 20 households per EA were selected, which provided a sample of 1000 households in each state. The EAs were selected from the National Integrated Survey of Households master sample for each state. Based on this application, the total sample size was 37 000 households.

The prevalence of zero-dose and underimmunized children aged 12 to 23 months varied significantly by region (geopolitical zone) and ethnicity (Table 1, Figure 3). In the Northern region, there is a higher percentage of children who either have not received any immunization (zero dose) or are not fully immunized (underimmunized)—specifically in the North West, where 45% of children have not received any immunizations and 57.5% are underimmunized. The states of Sokoto, Bauchi, and Zamfara in the North have the highest proportions of children who lack immunization, with Sokoto having 72.4% zero-dose and 88.5% underimmunized children, Bauchi having 57.7% and 69.7%, and Zamfara having 55.7% and 75.2%. Yet, the Southern region, particularly Ebonyi and Enugu states, exhibits a more positive immunization scenario, with a lower prevalence of children having received no immunization (1% zero dose) and being underimmunized (1.3%). Fulani households exhibit a higher prevalence of zero-dose and underimmunized children, with 53.5% having received no immunization and 69% being underimmunized. Similarly, among Hausa households, there is a notable occurrence of zero-dose and underimmunized children, with 46.6% having received no immunization and 58.8% being underimmunized (Table 1).

## DISCUSSION

The Immunization Agenda 2030 (IA2030) aims to half the children population that does not receive any DTP vaccine, with the ambitious objective to extend immunization services to zero-dose and underimmunized children and communities [1]. This analysis presents the observed prevalence of zero-dose and underimmunized children at the state level and the predicted prevalence at the third administrative area (LGA/district). We found that most zero-dose children in Nigeria are living in northern parts of the country, albeit with large variations across LGAs. Although the spatial distribution of underimmunized children is heterogeneous, the highest prevalence remains in the northern parts. These zero-dose and underimmunized children are among socially disadvantaged groups, including families in the lower wealth index quintiles and mothers with less education. Our findings also suggest that Nigeria will most likely not meet the IA2030 zero-dose target unless special accelerated efforts are made.

Multiple efforts have been implemented in Nigeria toward identifying the zero dose children and implementing priority interventions to address RI gaps. This includes the setup of the National Emergency Routine Immunization Coordination Centre (NERICC) in 2017, the introduction of new vaccines (eg, meningitis A, rotavirus vaccines, second dose of measles vaccine) into the national immunization schedule in 2019 and 2022, RI system-strengthening programs, and a zero-dose reduction plan implemented for RI and supplementary immunization activities in 2021 and 2022. Other efforts toward improving primary health care (PHC) delivery, such as the PHC Under One Roof, Basic Health Care Provision Fund, and the 2018–2028 Nigeria Strategy for Immunization and PHC Systems Strengthening, are expected to have direct and indirect impacts on the achievement of the IA2030 goals/objectives [18, 19]. Risk factors for zero-dose and underimmunized children have been widely documented in Nigeria [20, 21].

While RI remains the mainstay through which children receive their vaccinations, identifying areas with a high proportion of zero-dose and underimmunized children and targeting them is a key way of improving herd immunity and thereby preventing outbreaks.

Use of household surveys relies on sampling proportionate to estimated size to select clusters to be interviewed. Sparsely populated settlements, where zero-dose children are likely to be found, are less likely to be represented in surveys. Furthermore, insecure and difficult-to-reach areas, which may have a higher prevalence of zero-dose children, are often left out of surveys altogether. This selection bias may lead to situations where the prevalence of zero-dose and underimmunized children is underreported in many household surveys, including the MICS [10, 22]. Geostatistical techniques may help bring out remote areas and hence assist in the computation of the actual number of these children [14, 15, 17].

The use of geostatistical models to estimate the prevalence of zero-dose children, underimmunized children, missed communities, and other public health indicators opens opportunities for crafting interventions at a local scale and addressing issues before they escalate. In 2020, geospatial modeling was used to identify areas in Liberia that were at high risk during the Ebola virus outbreak (ie, hotspots of reported deaths). This information was used to effectively and efficiently respond to epidemics [23]. The models allow for the estimation of coverage at lower administrative levels, where data collection in these units would be a financial and logistical impossibility when conducted nationally in many countries. When applied temporally, the models help in monitoring and evaluating the impact of interventions over time. Cutts et al illustrated how information from vaccination coverage, measles incidence, and/or demographic/serologic data can estimate the spatial and age-specific distribution of measles susceptibility [22, 24]. The utility of the prediction, however, is as good as the quality of covariates publicly available to conduct frequent analysis.

This study has some limitations. First, there is currently a dearth of publicly available and updated covariates, and there is a need to invest in their generation. Second, the survey data used for this analysis are derived from the MICS/NICS, a national household-based survey that sources vaccination information from home-based vaccination records and maternal recall. With low rates of retention of home-based vaccination records in Nigeria, the likelihood of recall bias is high, especially in the context of an increasingly complex vaccination schedule. Third, while the model provides estimates for all LGAs, MICS/NICS and other household surveys exclude clusters in areas that had insecurity, which may also be areas with the largest burden of zero-dose and underimmunized children. Finally, the model makes use of geocoded data from publicly available surveys. These geocode data are usually anonymized by randomly displacing the location of the data collection point by 2 to 10 km from the original collection point before the data are released for secondary analysis. The effect of the anonymization on estimates has not been quantified. While there are limitations on the data used for this analysis, future investments in technology and training can aid in mitigating some or all these challenges.

The COVID-19 pandemic had an impact on the 2021 Nigeria MICS/NICS. The survey was conducted in 2 phases, with the first taking place in 2020 and the second in 2021. The pandemic caused delays in survey implementation, as well as changes to the survey methodology. For example, the survey team had to take extra precautions to protect the health of the respondents and the survey staff. This included conducting interviews over the phone or through video conferencing rather than in person. The survey team also had to make changes to the way that it collected data, such as using different forms and questionnaires. However, despite these challenges, the 2021 Nigeria MICS/NICS collected valuable data on the impact of the pandemic on children and families in Nigeria. The survey found that the pandemic had a significant impact on child health, education, and nutrition. For example, it revealed that the number of children who were not vaccinated against measles increased by 10% during the pandemic [10].

In conclusion, Nigeria—having more zero-dose and underimmunized children than any other country in the world—is in urgent need to strengthen its immunization system and increase coverage to protect the population. Geospatial modeling can help design targeted activities to deliver vaccines to underserved communities. This can be done by examining factors such as population density, access to health care, and socioeconomic status. Geospatial modeling can also be used to plan vaccine delivery routes that will efficiently reach underserved communities by considering factors such as the location of vaccination centers, the distance between communities, and the availability of transportation. By using geospatial modeling, countries can target their vaccine delivery efforts to underserved communities and ensure that all have access to the vaccines that they need.

## Notes

***Ethics approval and consent to participate***. The analysis for this study was conducted with secondary data, which do not necessitate consulting an ethics committee or obtaining consent to participate.

***Financial support*.** No financial support was received for this work. Funding to pay the Open Access publication charges for this article was provided by the authors.
